# Nurses' Attitudes Toward Innovative Neurotherapies in Memory Disorders: A Pilot Study

**DOI:** 10.1002/cns.71015

**Published:** 2026-07-17

**Authors:** Sini Huoponen, Juho Kettunen, Signe Mežinska, Ari Haaranen, Tarja Malm, Jukka Jolkkonen

**Affiliations:** ^1^ A.I.Virtanen Institute for Molecular Sciences University of Eastern Finland Kuopio Finland; ^2^ School of Computing University of Eastern Finland Joensuu Finland; ^3^ Institute of Clinical and Preventive Medicine University of Latvia Riga Latvia; ^4^ Department of Nursing Science University of Eastern Finland Kuopio Finland

**Keywords:** attitudes, expectations, innovative neurotherapies, memory disorders, nurses

## Abstract

**Background:**

As the aging population increases, the prevalence of Alzheimer's disease and other memory disorders increases, and emerging neurotherapies offer new hope, yet little is known about nurses' knowledge and perspectives on these innovations, despite their critical role in patient care. This study aimed to investigate nurses' attitudes, expectations, concerns, and perspectives toward innovative therapies for memory disorders.

**Methods:**

A multiple‐choice questionnaire (24 items) in Finnish was developed to explore nurses' views. The questionnaire was distributed via email to nursing homes in the Wellbeing Services County of North Savo, Finland, in autumn 2024. Composite scores for perceived benefits, perceived barriers, enabling factors, professional role‐related responsibilities, and worries were generated for statistical analyses.

**Results:**

A total of 132 nurses responded to the survey, most of whom were practical nurses working in nursing homes. Overall familiarity with innovative neurotherapies was low, and many participants indicated that they were not at all familiar with these treatments. Opinions on the future role of such therapies in managing memory disorders varied, with some considering them significant or moderately important. Several respondents also expressed serious concerns regarding the efficacy and potential side effects of these therapies. Regression analyses showed that lower composite outcome scores were consistently associated with being a social and healthcare student, being somewhat familiar with innovative neurotherapies, and, in some models, with shorter work experience or specific workplace settings. However, adjusted R^2^ values ranged from 0.09 to 0.18, indicating that demographic and professional background factors explained only a modest proportion of variance in the composite outcomes.

**Conclusion:**

Nurses currently have limited familiarity with innovative neurotherapies, but there is cautious optimism about their future role in treating memory disorders, including Alzheimer's disease.

## Graphical abstract

The aging population is increasing the prevalence of dementia, accelerating the development of emerging neurotherapies and their progression into early clinical trials. Nurses' attitudes, perceptions, and recognition of the importance of these therapies play a critical role in their successful implementation, making targeted education and training essential for effective integration into clinical practice. Graphical Abstract was generated with ChatGPT.

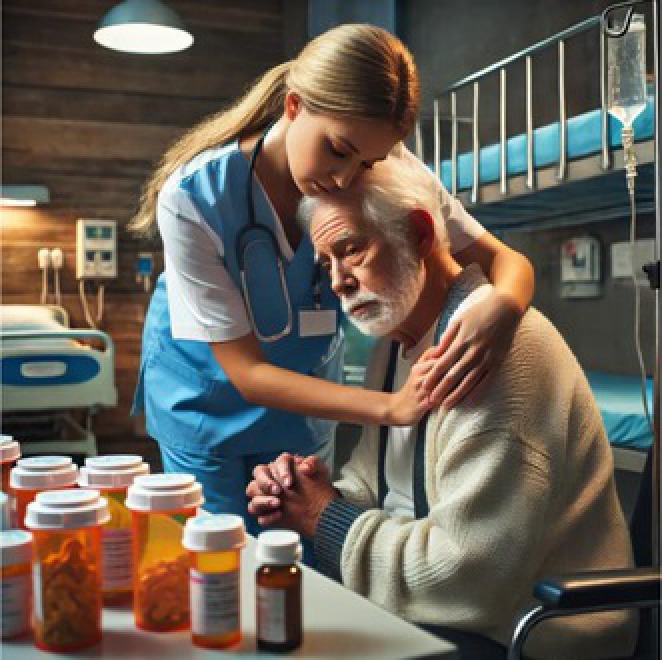

## 1 Introduction

As the population ages, the number of individuals affected by Alzheimer's disease (AD) and other memory disorders is expected to rise significantly, placing a substantial burden on society [1]. Unfortunately, there are currently no preventive or protective therapies available. However, innovative treatments for memory disorders are emerging, offering renewed hope to patients and their families. Several of these therapies are already in early‐phase clinical trials [2, 3]. Once available, the success of their clinical implementation will depend on the attitudes and engagement of all stakeholders, including nurses.

Nurses spend a considerable amount of time with patients and are uniquely positioned to understand and evaluate the impact and effectiveness of medical treatments. Despite their central role in patient care, evidence on nurses' familiarity with, expectations of, concerns about, and advocacy roles related to emerging advanced neurotherapies remains limited. Yet, nurses are well‐suited to be key collaborators in discussions about neuroscientific and neurotechnological progress [4]. Their ability to take on this role is closely tied to their professional education. In Finland, healthcare education is divided into registered nurse and practical nurse pathways, both of which emphasize ethical practice, lifelong learning, and interprofessional collaboration. However, there is currently no evidence on how these educational pathways address emerging therapies, such as innovative neurotherapies, leaving an important gap that warrants further investigation.

To fully realize the potential of advanced neurotherapies in improving the care of elderly people with memory disorders, it is essential to understand and incorporate nurses' views. As frontline caregivers and patient advocates, their insights are crucial for shaping more inclusive, ethical, and effective clinical translation of new treatments. The aim of this study is to explore nurses' familiarity with advanced neurotherapies, including their expectations, concerns, and perspectives. We analyzed the relationships between questionnaire responses and examined factors associated with composite variables related to innovative neurotherapies.

## 2 Methods

### 2.1 Study Design

This was a cross‐sectional, web‐based study conducted anonymously. Participation was entirely voluntary, and informed consent was obtained at the beginning of the questionnaire. Participants were informed that they would receive a summary of the study results. All collected data were treated confidentially and were accessible only to the research team.

### 2.2 Questionnaire

The questionnaire was in Finnish and was distributed via email to administrators of public (*n* = 52) and private (*n* = 43) nursing homes in the Wellbeing Services County of North Savo. Data collection took place between September and December 2024.

A multiple‐choice questionnaire (24 items) in Finnish was developed to address the objectives of the study (Supporting Information 1, English translation). The questionnaire was based on that used by Mosconi et al. [5]. and was further refined through a literature search conducted using the PubMed, Scopus, and CINAHL databases. Search terms included “nurses,” “advanced or innovative therapies,” and “Alzheimer's disease or memory disorders.” The questionnaire consisted of four sections: (1) background information, (2) benefits and expectations, (3) risks and concerns, and (4) nurses' role in shared decision‐making.

To ensure a consistent understanding among respondents, the questionnaire included definitions of key terms. *Memory disorders* were defined as Alzheimer's disease, cerebrovascular memory disorders, small or large vessel diseases of the brain, Lewy body‐related conditions, and frontotemporal degeneration. *Innovative neurotherapies* were defined as targeted drugs intended to stop or slow disease progression, cell therapies, immunotherapies, and other non‐pharmacological treatments.

A small‐scale pilot test was conducted in August 2024 at a private nursing home (Mainiokoti Metsänhelmi, Kuopio). Based on the feedback received, the questionnaire was revised as necessary (e.g., simplified wording for clarity) and subsequently administered electronically.

### 2.3 Statistical Analysis

All statistical analyses were performed in R version 4.4.1 [6]. Power analysis was conducted using Cohen's effect size $f^{2}$ to estimate the required sample size for multiple linear regression. Assuming a statistical power of 80%, a medium effect size ($f^{2}=0.15$), a significance level of α = 0.05, and 12 predictors, the analysis indicated that a minimum sample size of approximately 130 participants would be required.

Associations between categorical variables were tested using Fisher's exact test. For contingency tables larger than 2 × 2 where exact computation of the *p*‐value is computationally intensive, *p*‐values were estimated via Monte Carlo simulation using the *fisher.test()* function in R. All tests were two‐sided, and a significance level of α = 0.05 was applied.

Composite variables were constructed by averaging Likert‐scale items representing conceptually related constructs: Perceived benefits (5 items), perceived barriers (13 items), enabling factors (10 items), professional role‐related responsibilities (7 items), and worries (2 items) regarding innovative neurotherapies (Supporting Information 2). The composite variables were based on theoretically and conceptually related questionnaire items derived from the survey structure and previous literature. Internal consistency of each composite variable was assessed using Cronbach's alpha. All composite variables demonstrated good to excellent internal consistency. Cronbach's alpha values were α = 0.89 for perceived benefits, α = 0.91 for perceived barriers, α = 0.92 for enablers, α = 0.88 for professional role‐related responsibilities, and α = 0.85 for worries.

Prior to aggregation, item responses were recoded into numeric values, and missing values were treated as missing. The worry composite was calculated only from substantive Likert‐type responses. “I don't know” responses were excluded by coding them as missing values. Composite scores were calculated as the mean of item responses only for respondents with complete data on all items within each scale. All items were coded on a five‐point Likert scale (1–5) with higher values indicating greater perceived importance or agreement.

Spearman correlation coefficients were calculated to assess associations between demographic factors and composite variables.

Linear regression models were used to examine factors associated with each composite outcome (i.e., benefits, barriers, enablers, professional role‐related responsibilities, and worries). Candidate predictors included age, sex, education, workplace, work experience, and familiarity with innovative neurotherapies. Initial regression models included all candidate predictors after which backward elimination was used to remove non‐significant variables and obtain the final models, with predictors retained at a significance threshold of *p* < 0.10 to allow identification of potentially meaningful explanatory variables. Because composite variables consisted of multiple items and demonstrated good internal consistency, the scores were treated as approximately continuous variables in the regression analysis. Linearity, homoscedasticity, multicollinearity, and normality of residuals assumptions were assessed, and no major violations were observed. Linearity and homoscedasticity were evaluated using residuals vs. fitted values plots, and normality of residuals was assessed using Q–Q plots. Multicollinearity was assessed using variance inflation factors (VIF). Across the models, the unadjusted GVIF values ranged from 1.02 to 2.36, and adjusted GVIF values, calculated as GVIF ^1/(2 × Df)^, ranged from 1.00 to 1.11, indicating no evidence of multicollinearity in any of the models.

## 3 Results

### 3.1 Demographic and Professional Characteristics of Survey Respondents

A total of 132 individuals responded to the survey (response rate: approximately 10%). Most were female (*n* = 121, 91.7%), with 10 (7.6%) identifying as male and one selecting “other/prefer not to say.” Participants' ages ranged from under 20 years (*n* = 1, 0.8%), 20–30 years (*n* = 13, 9.1%), 30–40 years (*n* = 23, 17.4%), and 40–50 years (*n* = 35, 26.5%), with the majority being over 50 years old (*n* = 60, 45.5%). Age was significantly associated with specialization (*p* < 0.05), work experience (*p* < 0.001), and familiarity with innovative neurotherapies (*p* < 0.05). The aging workforce and upcoming retirements pose a substantial challenge for the near future.

Most respondents were qualified as practical nurses (*n* = 86, 65.2%). Other backgrounds included registered/public health nurses (*n* = 23, 17.4%), University of Applied Sciences (UAS) degrees in health sciences (*n* = 9, 6.8%), social and health care students (*n* = 6, 4.5%), and other qualifications (*n* = 8, 6.1%). Associations between basic training and other variables are shown in Table 1.

**TABLE 1:** Associations between basic training and other variables.

Basic training	
Age	0.025
Specialization	0.032
Work experience	0.000
Scientific research and development	0.022
Media ethics and appropriateness of information	0.030
Limited number of nursing staff	0.018
Patient resistance or negative attitudes	0.035
Family and community opposition or negative attitudes	0.012

*Note:* Associations were analyzed using Fisher's exact test; *p*‐values are shown.

Most respondents were employed in nursing homes (*n* = 117, 88.6%). Other reported workplaces included service or group homes (*n* = 11, 8.3%), home care (*n* = 1, 0.8%), and other unspecified settings (*n* = 3, 2.3%). In addition, 91 (68.9%) respondents reported working in the public social and health care sector, while 41 (31.1%) were employed in the private social and health care sector. Most nurses were experienced professionals. Work experience ranged from less than one year (*n* = 6, 4.5%), 1–5 years (*n* = 24, 18.2%), 5–10 years (*n* = 25, 18.9%), 10–20 years (*n* = 29, 22.0%) and majority (*n* = 48, 36.4%) reporting to have 20 or more years of experience.

### 3.2 Knowledge and Perceptions

**FIGURE 1:**
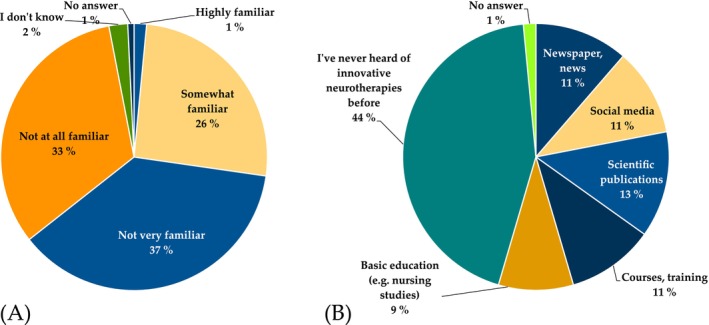
Nurses' familiarity with innovative neurotherapies for memory disorders (A) and their sources of information (B).

Nurses' familiarity with innovative neurotherapies varied (Figure 1A): 43 (32.6%) respondents reported being not at all familiar, 49 (37.1%) not very familiar, 34 (25.8%) somewhat familiar, and 2 (1.5%) highly familiar. Additionally, 3 (2.3%) respondents answered “I don't know,” and 1 (0.8%) did not respond to the question. There was a significant relationship between age and how familiar the participants were with innovative neurotherapies for memory disorders (*p* < 0.05).

Sources of information about innovative neurotherapies included scientific publications (*n* = 17, 12.9%), newspapers (*n* = 15, 11.4%), social media (*n* = 14, 10.6%), courses and training (*n* = 14, 10.6%), and basic education, such as nursing studies (*n* = 12, 9.1%). The majority of respondents (*n* = 58, 43.9%) reported that they had never heard of innovative neurotherapies before (Figure 1B).

When asked about the perceived future significance of innovative neurotherapies in treating memory disorders, nurses rated their potential as significant (*n* = 53, 40.2%), moderate (*n* = 59, 44.7%), or uncertain (*n* = 11, 8.3%), with smaller numbers considering them minor (*n* = 4, 3.0%) or not feasible (*n* = 5, 3.8%). The following patient‐centered outcomes were most often rated as critically important: Improved cognitive functions (*n* = 72, 54.5%), improved functional ability (*n* = 75, 56.8%), reduced behavioral symptoms (*n* = 81, 61.1%), improved quality of life (*n* = 96, 72.7%), and enhanced family satisfaction (*n* = 46, 34.8%). Respondents identified critical factors for successful clinical translation, including patient and family engagement (*n* = 59, 44.7%), multidisciplinary collaboration (*n* = 81, 61.4%), scientific research (*n* = 78, 59.1%), adequate resources (*n* = 94, 71.2%), and training (*n* = 93, 70.4%).

### 3.3 Potential Risks Associated With Innovative Neurotherapy

**FIGURE 2:**
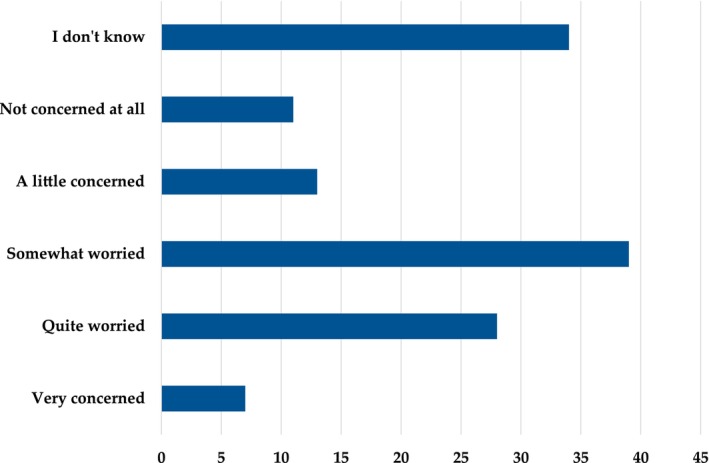
Nurses' concerns about the potential lack of efficacy of innovative neurotherapies. Numbers indicate responses (n).

**FIGURE 3:**
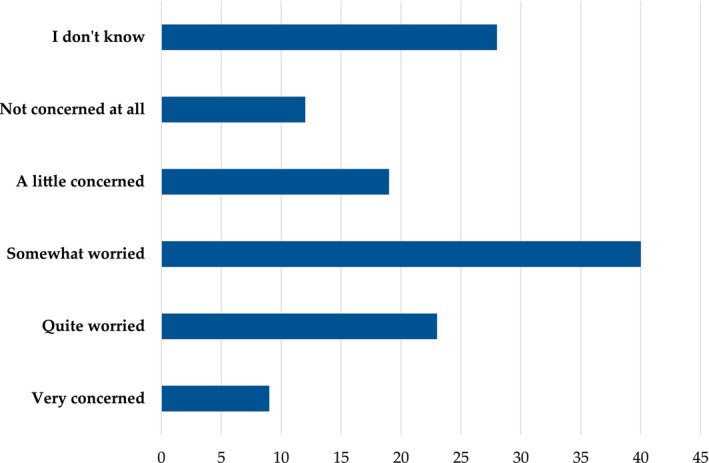
Nurses' concerns about the potential side effects of innovative neurotherapies. Numbers indicate responses (n).

When asked about the potential lack of therapeutic efficacy, 7 (5.3%) nurses reported being *very concerned*, 28 (21.2%) were *quite worried*, and 39 (29.5%) were *somewhat worried* (Figure 2). A similar pattern was observed regarding concerns about side effects: 9 (6.8%) nurses were *very concerned*, 23 (17.4%) were *quite concerned*, and 40 (30.3%) were *somewhat concerned* (Figure 3). A high number of nurses also responded with “I don't know.”

Fisher's Exact Test revealed a significant association between concerns about therapeutic efficacy and age (*p* < 0.05). Significant associations were also found between concerns about side effects and (1) familiarity with innovative neurotherapies (*p* < 0.05) and (2) the source through which information about neurotherapies was received (*p* < 0.05).

### 3.4 Correlations Between the Demographic Factors and Composite Variables

Spearman's rank order correlation coefficients showed that age and work experience were not significantly associated with the composite variables (*ρ* = −0.02–0.15, *p* > 0.05) (Table 2). Familiarity with innovative neurotherapies showed weak negative correlations with perceived benefits (*ρ* = −0.20, *p* = 0.024), enablers (*ρ* = −0.19, *p* = 0.037), and barriers (*ρ* = −0.18, *p* = 0.042) but not with professional role‐related responsibilities or worry. Age and work experience were moderately correlated (*ρ* = 0.51, *p* < 0.001) while familiarity with innovative neurotherapies showed a weak negative correlation with work experience (*ρ* = −0.29, *p* = 0.001).

**TABLE 2:** Spearman**'**s correlation coefficients between age, familiarity, work experience, and composite variables.

Variable	Age	Familiarity	Work experience	Benefits	Enablers	Barriers	Tasks	Worry
**Age**	—	−0.12	0.51	0.06	0.08	0.03	0.11	0.15
		NS	*p* < 0.001	NS	NS	NS	NS	NS
**Familiarity**	−0.12	—	−0.29	−0.20	−0.19	−0.18	−0.13	0.09
	NS		*p* < 0.001	*p* = 0.024	*p* = 0.037	*p* = 0.042	NS	NS
**Work experience**	0.51	−0.29	—	0.10	0.10	0.14	0.10	−0.02
	*p* < 0.001	*p* < 0.001		NS	NS	NS	NS	NS
**Benefits**	0.06	−0.20	0.10	—	0.62	0.46	0.48	0.27
	NS	*p* = 0.024	NS		*p* < 0.001	*p* < 0.001	*p* < 0.001	*p* < 0.01
**Enablers**	0.08	−0.19	0.10	0.62	—	0.64	0.68	0.23
	NS	*p* = 0.037	NS	*p* < 0.001		*p* < 0.001	*p* < 0.001	*p* = 0.019
**Barriers**	0.03	−0.18	0.14	0.46	0.64	—	0.57	0.40
	NS	*p* = 0.042	NS	*p* < 0.001	*p* < 0.001		*p* < 0.001	*p* < 0.001
**Tasks**	0.11	−0.13	0.10	0.48	0.68	0.57	—	0.33
	NS	NS	NS	*p* < 0.001	*p* < 0.001	*p* < 0.001		*p* < 0.001
**Worry**	0.15	0.09	−0.02	0.27	0.23	0.40	0.33	—
	NS	NS	NS	*p* < 0.01	*p* = 0.019	*p* < 0.001	*p* < 0.001	

Abbreviation: NS, not significant.

Moderate positive associations were found between the composite variables (Table 2). The strongest correlations were observed between enablers and professional role‐related responsibilities (*ρ* = 0.68, *p* < 0.001), enablers and barriers (*ρ* = 0.64, *p* < 0.001), and benefits and enablers (*ρ* = 0.62, *p* < 0.001). Benefits were also moderately correlated with professional role‐related responsibilities (*ρ* = 0.48, *p* < 0.001) and barriers (*ρ* = 0.46, *p* < 0.001), while barriers and professional role‐related responsibilities showed a moderate association (*ρ* = 0.57, *p* < 0.001) and with worries (*ρ* = 0.40, *p* < 0.001). Worries were also positively correlated with professional role‐related responsibilities (*ρ* = 0.33, *p* < 0.001), benefits (*ρ* = 0.27, *p* = 0.006), and enablers (*ρ* = 0.23, *p* = 0.019).

### 3.5 Regression Analysis Identifying Factors Associated With Composite Outcome Scores

Across regression models, being a social and healthcare student and being somewhat familiar with innovative neurotherapies were consistently associated with lower scores for perceived benefits, barriers, and enabling factors, indicating lower perceived importance or agreement in these domains. Working in service housing/group home settings was additionally associated with lower enabling factor scores. Lower professional role‐related responsibility scores were associated with being a social and healthcare student and with longer work experience compared with the reference category (< 1 year). Similarly, lower worry scores were associated with longer work experience compared with the reference category (< 1 year) and student status. All models were statistically significant except the worries model, which showed borderline non‐significance (*p* = 0.057). Therefore, the individual predictor effects in the worries model should be interpreted cautiously. Adjusted R^2^ values ranged from 0.09 to 0.18. Full regression model estimates are presented in Table S1.

## 4 Discussion

Nurses play a critical role in caring for elderly patients, including those with Alzheimer's disease and other forms of dementia. While previous research has examined nurses' knowledge and attitudes toward geriatric patients [7, 8, 9] and nurses' perspectives on dementia prevention [10], little is known about their expectations regarding treatment—particularly the adoption of emerging neurotherapies. To our knowledge, this is the first study to explore nursing home nurses' attitudes toward innovative neurotherapies. By examining this previously unexplored area of nurses' knowledge, expectations, and concerns, we can gain valuable insights into their pivotal role in patient care and in shaping acceptance of new treatments. Understanding nurses' perspectives can also inform tailored strategies that strengthen patient engagement, improve treatment adherence, and enhance overall quality of care—ultimately leading to better treatment experiences and outcomes for patients.

### 4.1 Familiarity With Innovative Neurotherapies Was Low

We found that nurses' familiarity with innovative neurotherapies was generally low and varied across respondents, with age playing a significant role. Nurses over 50 years old were particularly less familiar with these approaches.

Consistent with this finding, familiarity with innovative neurotherapies was weakly negatively correlated with work experience, indicating that less experienced respondents reported slightly greater familiarity than more experienced professionals. One possible explanation is that recent educational curricula may place greater emphasis on emerging technologies and novel therapeutic approaches, resulting in newer entrants to the profession being more exposed to innovative neurotherapies during training.

Supporting this interpretation, healthcare students, respondents reporting some familiarity with innovative neurotherapies, and nurses with shorter work experience tended to assign lower importance to perceived benefits, barriers, enabling factors, and professional role‐related responsibilities. This pattern may suggest that individuals with less professional experience or more recent educational exposure approach innovative neurotherapies from a more neutral or less polarized perspective, perceiving both potential advantages and implementation‐related considerations as less pronounced.

### 4.2 Nurses' Attitudes Toward Innovative Neurotherapies

There was cautious optimism among nurses regarding the future role of neurotherapies. Although the broader literature remains limited, findings from related fields provide useful context. For example, Du et al. found that older age and higher education level were associated with higher knowledge, attitude, and/or practice scores [11]. Similarly, another Chinese study by Wang et al. reported that despite limited understanding of Alzheimer's disease, nurses held positive attitudes toward its care [12]. These findings help contextualize our results within an international framework while highlighting the paucity of literature specifically focused on neurotherapies.

Related studies from other clinical domains have likewise examined nurses' knowledge and attitudes toward medical interventions. A cross‐sectional survey by Yu et al. found that physicians and nurses generally demonstrated strong knowledge, positive attitudes, and appropriate practices regarding continuous renal replacement therapy, with competence primarily acquired through training and clinical experience [13].

Similarly, research on cancer clinical trials has shown that nurses generally hold positive attitudes toward experimental treatments, although perceptions vary according to work setting, professional role, experience, and education level. Oncology nurses emphasized that experimental treatments should demonstrate clear and measurable benefits before implementation [14]. In addition, Hayek et al. found that improving nurses' knowledge and emphasizing the benefits of early palliative care can foster more positive attitudes, while investment in comprehensive education and training helps ensure high‐quality care, improves patient outcomes, and reduces suffering [15].

### 4.3 Efficacy and Safety Are Major Concerns

Concerns about efficacy and safety emerged unexpectedly from the nurses' responses. Most reported being somewhat, quite, or very concerned about a potential lack of therapeutic benefit, and similar concerns were raised about possible side effects. As these are common reasons for terminating clinical trials [16], this might account for the nurses' cautious expectations toward innovative therapies. Social media, as discussed later, may also raise unnecessary concerns. Another possible reason could be the already high workload, limited resources, and insufficient personnel available to implement new treatments [17]. Concerns may also reflect generalized medical skepticism rather than risks specifically associated with neurotherapies, particularly among respondents who reported being ‘not at all familiar’ with these treatments. At the same time, less experienced individuals appeared to report higher levels of concern in relation to innovative neurotherapies, whereas more experienced respondents reported lower levels of worry. This may reflect uncertainty or lower confidence among less experienced professionals when considering unfamiliar or technologically advanced interventions. However, because the association between work experience and worry emerged from a modest sample and the overall model for worries was only borderline statistically significant, these findings should be interpreted cautiously.

### 4.4 Importance of Continuous Training

Many nurses had never heard of these treatments, while others reported learning about them primarily through newspapers and social media. Although the media plays an important role in shaping public understanding of Alzheimer's disease, it can also amplify premature optimism and foster unrealistic expectations [18]. A recent Norwegian study further showed that nurses generally possess insufficient knowledge in pharmacology [19]. These gaps suggest not only a need for continuous professional education among practicing nurses but also for nursing students and their educators to receive targeted training on innovative neurotherapies to ensure preparedness for future clinical practice [9]. In a small semi‐structured study, Deloria and Wolbring found that participants believed nurses could meaningfully contribute to discussions on the governance of neuroscientific and neurotechnological advancements—provided they were offered greater opportunities and access to lifelong learning [4]. Despite limited familiarity, nurses acknowledged the potential of innovative therapies to enhance cognitive function and improve family satisfaction. They identified multidisciplinary collaboration, ongoing research, adequate resources, and targeted training as essential for integrating these treatments into routine care. Coordinated efforts among physicians, nurses, therapists, social workers, and other providers can not only elevate care quality but also reduce caregiver burden and enhance patients' quality of life [20].

### 4.5 Limitations

We acknowledge several limitations of the present study. The study was conducted in the North Savo region in Finland, and with a relatively small sample size, underscoring the need for international research with a broader participant base to enhance the robustness and generalizability of the findings.

Regarding sample size considerations, previous methodological literature suggests that a minimum sample of approximately 100 respondents is acceptable for multivariable regression analyses [21]. In support of this, our power analysis indicated that a minimum sample size of approximately 130 participants would be required. Furthermore, the demographic characteristics of our sample (e.g., sex, age, and education) are broadly consistent with those reported for the Finnish nursing workforce, suggesting a reasonable level of representativeness. Nevertheless, we acknowledge that these considerations do not fully eliminate concerns regarding generalizability.

The modest adjusted R^2^ values observed in the regression models should also be considered when interpreting the findings. Although demographic and professional background factors were associated with the composite outcomes in the regression analyses, they explained only a modest proportion of the variance in nurses’ attitudes. This suggests that factors not measured in this study, such as personal beliefs, previous experiences with medical innovations, and institutional culture, may play a larger role in shaping nurses’ attitudes toward innovative neurotherapies.

Nurses’ familiarity with innovative neurotherapies varied, which may have introduced bias. To avoid misclassifying lack of knowledge as a neutral attitude, responses of “I don't know” were treated as missing values rather than neutral responses. In addition, the high number of “I don't know” responses suggests that some questions may have been too challenging or not clearly understood. “I don't know” responses also indicate that participation was not limited to individuals with a pre‐existing interest in the topic and thereby reduce the likelihood of significant selection bias.

To promote a shared understanding, the questionnaire also included brief definitions of innovative neurotherapies. Due to space limitations, more detailed explanations (e.g., mechanisms of action, delivery protocols, or specific adverse effects) could not be provided. We acknowledge that some responses may therefore reflect limited knowledge or conjecture. Nevertheless, limited knowledge does not preclude the formation of attitudes toward innovative neurotherapies. Attitudes may be based on beliefs, assumptions, or inferences even in the absence of comprehensive knowledge [22]. Accordingly, although some respondents reported limited familiarity with these therapies, their attitudes still provide insight into prevailing perceptions and openness toward such interventions.

## 5 Conclusions

Emerging neurotherapies for Alzheimer's disease and other memory disorders offer renewed hope through innovative approaches such as targeting underlying biological mechanisms with disease‐modifying drugs, employing non‐pharmacological technologies, and developing immunotherapies [23]. These advancements aim not only to relieve symptoms but also to potentially halt or reverse disease progression. Although many of these approaches are already in preclinical or early‐phase clinical trials, their complexity, uncertain efficacy, and potential side effects require careful consideration as suggested by the present study. Nurses’ input is critical to ensuring the safe, effective, and patient‐centered integration of emerging neurotherapies into clinical practice as they become available. To achieve this, additional training for nurses is essential to overcoming the challenges associated with innovative neurotherapies.

## Funding

This was an EU Joint Programme ‐ Neurodegenerative Disease Research (JPND) project funded through the Research Council of Finland, No. 357769.

## Ethics Statement

The study was approved by the Regional Medical Research Ethics Committee of the Wellbeing Services County of North Savo (approval date: August 7, 2024; reference number: 198/13.00/2024).

## Consent

Informed consent was obtained from all participants on the first page of the questionnaire. We adhered to strict data anonymization protocols to ensure participant confidentiality and privacy.

## Conflicts of Interest

The authors declare no conflicts of interest.

## Supporting information


**Supporting Information: 1** Questionnaire in English.

**Supporting Information: 2** Definitions of composite variables.

**Table S1:** Multiple linear regression analyses.

## Data Availability

The data that support the findings of this study are available on request from the corresponding author. The data are not publicly available due to privacy or ethical restrictions.
