# Supplementary material for: Nurses' Attitudes Toward Innovative Neurotherapies in Memory Disorders: A Pilot Study
Source: CNS Neurosci Ther. 2026 Jul 17;32(7):e71015. doi: 10.1002/cns.71015 (PMC13377797; doi:10.1002/cns.71015)
Supplement: Supplementary file 1 — Supporting Information: 1 Questionnaire in English. [file CNS-32-e71015-s002.docx]

**Nurses and innovative neurotherapies in memory disorders**

The questionnaire was based on that used by Mosconi *et al*. [2023] and was further refined through a literature search

conducted using the PubMed, Scopus, and CINAHL databases. Search terms included “nurses,” “advanced or innovative therapies,” and “Alzheimer’s disease or memory disorders.”

Mosconi P, Colombo C, Paletta P, Gangeri L, Pellegrini C, Garralda E, et al. Public and patient involvement: a survey on knowledge, experience and opinions among researchers within a precision oncology European project. BMC Cancer 2023

**Important definitions**

**Memory disorders** = Alzheimer's disease, memory disease of cerebrovascular disease, disease of small or large veins of the brain, diseases related to Lewy body pathology, frontal lobe degeneration

**Innovative neurotherapies** = targeted drugs aimed at stopping or slowing down the disease, stem cells, vaccines and other non-pharmacological treatments

**Involvement** = involving the patient in decision-making related to their own care

**Part 1**

**Nurses and innovative neurotherapies in memory disorders**

**Background information**

1. **Consent**

- I confirm my participation in this study

1. **Gender**

- Man
- Female
- Other/I don't want to report

1. **Age**

- <20 years
- 20-30 years
- 40-50 years
- >50 years

1. **Basic training (if multiple, select the highest completed degree)**

- Student
- Practical nurse
- Nurse or public health nurse
- Master of Health Sciences/Master of Science
- Other education

1. **Specialization (if applicable)**

- Surgical treatment
- Intensive care
- Geriatric nursing
- Psychiatric nursing
- Other option

1. **Current working environment (North Savo Welfare Area)**

- Public social and health services
- Private social and health services
- Other work environments (e.g. gig work)

1. **Current job (North Savo Welfare Area)**

- Medical center
- Service house/group home
- Care home (round-the-clock care)
- Home care
- Own business
- Other

1. **Work experience**

- < year
- 1-5 years
- 5-10 years
- 10-20 years
- >20 years

1. **How familiar are you with innovative neurotherapies for memory disorders?**

- Highly familiar
- Somewhat familiar
- Not very familiar
- Not at all familiar
- I don't know

1. **Through which of the following sources have you received your information about innovative neurotherapies?**

- Newspaper, news
- Social media
- Scientific publications
- Courses, training
- Basic education (e.g. nursing studies)
- I've never heard of innovative neurotherapies before

**Part 2**

**Nurses and innovative neurotherapies in memory disorders**

**What benefits do you expect innovative neurotherapies to provide?**

**Innovative neurotherapies** = targeted drugs aimed at stopping or slowing down the disease, stem cells, immunotherapies and other non-pharmacological treatments

1. **How significant role do you think innovative neurotherapies will play in treating memory disorders in the future?**

- Significant
- Moderate
- Minor
- Uncertain
- I don't think they're feasible in a normal nursing job/no role

1. **When assessing the effectiveness of innovative neurotherapies, how important do you consider the following patient-centered outcomes?**

|  |  |  |  |  |  |  |  | Critically | Very | Moderately | Slightly | Not important |
| --- | --- | --- | --- | --- | --- | --- | --- | --- | --- | --- | --- | --- |
|  |  |  |  |  |  |  |  | important | important | important | important | at all |
| Preserving or improving cognitive functions | | | | |  |  |  | Ο | Ο | Ο | Ο | Ο |
| Preserving or improving functional ability | | | | |  |  |  | Ο | Ο | Ο | Ο | Ο |
| Reducing symptoms (e.g., behavioral symptoms) | | | | | |  |  | Ο | Ο | Ο | Ο | Ο |
| Improving quality of life | | |  |  |  |  |  | Ο | Ο | Ο | Ο | Ο |
| Enhancing family satisfaction | | | |  |  |  |  | Ο | Ο | Ο | Ο | Ο |

1. **To what extent do you believe the expectations of patients and their families will influence the successful implementation of innovative neurotherapies?**

- Significant role
- Moderate role
- Minor role
- No role
- I don't know

1. **How important do you consider the following factors to ensure that innovative neurotherapies could become part of practical patient work?**

|  |  |  |  |  |  |  |  | Critically | Very | Moderately | Slightly | Not important |
| --- | --- | --- | --- | --- | --- | --- | --- | --- | --- | --- | --- | --- |
|  |  |  |  |  |  |  |  | important | important | important | important | at all |
| Enhance nursing staff training and continuous professional development | | | | | | | | Ο | Ο | Ο | Ο | Ο |
| Ensure adequate resource allocation | | | | |  |  |  | Ο | Ο | Ο | Ο | Ο |
| Promote scientific research and development | | | | |  |  |  | Ο | Ο | Ο | Ο | Ο |
| Foster multidisciplinary and multiprofessional collaboration | | | | | | |  | Ο | Ο | Ο | Ο | Ο |
| Engage patients and their families in decision-making processes | | | | | | | | Ο | Ο | Ο | Ο | Ο |

1. **How important are the following factors to avoid unnecessary and excessive expectations of the benefits of innovative neurotherapy?**

|  |  |  |  |  |  |  |  | Critically | Very | Moderately | Slightly | Not important |
| --- | --- | --- | --- | --- | --- | --- | --- | --- | --- | --- | --- | --- |
|  |  |  |  |  |  |  |  | important | important | important | important | at all |
| Enhance specialized training for nursing staff on the topic | | | | | | |  | Ο | Ο | Ο | Ο | Ο |
| Foster general discussion to raise awareness | | | | | |  |  | Ο | Ο | Ο | Ο | Ο |
| Provide clear, accessible, and relevant information | | | | | |  |  | Ο | Ο | Ο | Ο | Ο |
| Emphasize media ethics and ensure appropriateness of information | | | | | | | | Ο | Ο | Ο | Ο | Ο |
| Define the roles and responsibilities of patient organizations | | | | | | |  | Ο | Ο | Ο | Ο | Ο |

**Part 3**

**Nurses and innovative neurotherapies in memory disorders**

**What are the potential challenges and risks associated with advanced neurotherapy?**

**Innovative neurotherapies** = targeted drugs aimed at stopping or slowing down the disease, stem cells, immunotherapies and other non-pharmacological treatments

1. **How important do you consider the following resource challenges to be as limitations to the use of advanced neurotherapies?**

|  |  |  |  |  |  |  |  | Critically | Very | Moderately | Slightly | Not important |
| --- | --- | --- | --- | --- | --- | --- | --- | --- | --- | --- | --- | --- |
|  |  |  |  |  |  |  |  | important | important | important | important | at all |
| Insufficient training for nurses | | | |  |  |  |  | Ο | Ο | Ο | Ο | Ο |
| Limited nursing staff numbers | | | |  |  |  |  | Ο | Ο | Ο | Ο | Ο |
| High turnover rates among nursing staff | | | | |  |  |  | Ο | Ο | Ο | Ο | Ο |
| Elevated costs | |  |  |  |  |  |  | Ο | Ο | Ο | Ο | Ο |

1. **How important do you consider the following attitude-related challenges in limiting the use of innovative neurotherapies?**

|  |  |  |  |  |  |  |  | Critically | Very | Moderately | Slightly | Not important |
| --- | --- | --- | --- | --- | --- | --- | --- | --- | --- | --- | --- | --- |
|  |  |  |  |  |  |  |  | important | important | important | important | at all |
| Patient resistance or negative attitudes | | | | |  |  |  | Ο | Ο | Ο | Ο | Ο |
| Opposition or negative attitudes from family and community | | | | | | |  | Ο | Ο | Ο | Ο | Ο |
| Prevailing negative public opinion and debate | | | | | |  |  | Ο | Ο | Ο | Ο | Ο |
| Stigmatization of memory disorders | | | |  |  |  |  | Ο | Ο | Ο | Ο | Ο |

1. **How concerned are you about the potential lack of efficacy or therapeutic effect of innovative neurotherapies?**

- Very concerned
- Quite worried
- Somewhat worried
- A little concerned
- Not concerned at all
- I don't know

1. **How concerned are you about the potential side effects of innovative neurotherapies?**

- Very concerned
- Quite concerned
- Somewhat concerned
- A little concerned
- Not concerned at all
- I don't know

1. **How important do you consider the following factors in slowing down the uptake of innovative neurotherapies?**

|  |  |  |  |  |  |  |  | Critically | Very | Moderately | Slightly | Not important |
| --- | --- | --- | --- | --- | --- | --- | --- | --- | --- | --- | --- | --- |
|  |  |  |  |  |  |  |  | important | important | important | important | at all |
| Insufficient research evidence | | | |  |  |  |  | Ο | Ο | Ο | Ο | Ο |
| Limited resources | | |  |  |  |  |  | Ο | Ο | Ο | Ο | Ο |
| Attitudinal obstacles and challenges | | | |  |  |  |  | Ο | Ο | Ο | Ο | Ο |
| Difficulties involving patients in decision-making about their treatment | | | | | | | | Ο | Ο | Ο | Ο | Ο |
| Inadequate consideration of patients' individual treatment needs | | | | | | |  | Ο | Ο | Ο | Ο | Ο |

**Part 4**

**Nurses and innovative neurotherapies in memory diseases**

**Nurses, innovative neurotherapy and inclusion of patients and loved ones**

**Involvement** = involving the patient in decision-making related to their own care

1. **Have you received specialized training in patient and close involvement?**

- Yes, extensive training
- Yes, some training
- Only a little training
- Not at all
- I don't know

1. **How important do you consider the involvement of patients and loved ones in patient care?**

- Very important
- Important
- Moderately important
- Not very important
- Not at all important

1. **How important is the information provided by nurses to patients and loved ones about new innovative neurotherapies?**

- Very important
- Important
- Moderately important
- Not very important
- Not at all important

1. **How important do you consider the following ways in which nurses can promote the use of innovative neurotherapies in the treatment of memory disorders?**

|  |  |  |  |  |  |  |  | Critically | Very | Moderately | Slightly | Not important |
| --- | --- | --- | --- | --- | --- | --- | --- | --- | --- | --- | --- | --- |
|  |  |  |  |  |  |  |  | important | important | important | important | at all |
| Support the patient | | |  |  |  |  |  | Ο | Ο | Ο | Ο | Ο |
| Support loved ones | | |  |  |  |  |  | Ο | Ο | Ο | Ο | Ο |
| Participate in training/update information | | | | |  |  |  | Ο | Ο | Ο | Ο | Ο |
| Monitor patients and evaluate/report treatment response | | | | | | |  | Ο | Ο | Ο | Ο | Ο |
| Working as part of a multidisciplinary team | | | | | |  |  | Ο | Ο | Ο | Ο | Ο |
