# Supplementary material for: Nurses' Attitudes Toward Innovative Neurotherapies in Memory Disorders: A Pilot Study
Source: CNS Neurosci Ther. 2026 Jul 17;32(7):e71015. doi: 10.1002/cns.71015 (PMC13377797; doi:10.1002/cns.71015)
Supplement: Supplementary file 2 — Supporting Information: 2 Definitions of composite variables. [file CNS-32-e71015-s003.docx]

Composite variables were constructed by averaging Likert-scale items representing conceptually related constructs perceived benefits, perceived barriers, enabling factors, professional role-related responsibilities and worries regarding innovative neurotherapies.

Prior to aggregation item responses were recoded into numeric values and missing values were treated as missing. The worry composite was calculated only from substantive Likert-type responses. "I cannot say" responses were excluded by coding them as missing values. Composite scores were calculated as the mean of item responses only for respondents with complete data on all items within each scale. All items were coded on a five-point Likert scale (1–5) with higher values indicating greater perceived importance or agreement.

**Perceived benefit score (question 12):**

Preserving or improving cognitive functions

Preserving or improving functional ability

Reducing symptoms (e.g., behavioral symptoms)

Improving quality of life

Enhancing family satisfaction

**Enabler factors score (questions 14 and 15):**

Enhance nursing staff training and continuous professional development

Ensure adequate resource allocation

Promote scientific research and development

Foster multidisciplinary and multiprofessional collaboration

Engage patients and their families in decision-making processes

Enhance specialized training for nursing staff on the topic

Foster general discussion to raise awareness

Provide clear, accessible, and relevant information

Emphasize media ethics and ensure appropriateness of information

Define the roles and responsibilities of patient organizations

**Perceived barrier score (questions 16, 17 and 20):**

Insufficient training for nurses

Limited nursing staff numbers

High turnover rates among nursing staff

Elevated costs

Patient resistance or negative attitudes

Opposition or negative attitudes from family and community

Prevailing negative public opinion and debate

Stigmatization of memory disorders

Insufficient research evidence

Limited resources

Attitudinal obstacles and challenges

Difficulties involving patients in decision-making about their treatment

Inadequate consideration of patients' individual treatment needs

**Professional role-related responsibilities score (questions 22-24):**

How important do you consider the involvement of patients and loved ones in patient care?

How important is the information provided by nurses to patients and loved ones about new innovative neurotherapies?

Support the patient

Support loved ones

Participate in training/update information

Monitor patients and evaluate/report treatment response

Working as part of a multidisciplinary team

**Worry_score (questions 18 and 19):**

How concerned are you about the potential lack of efficacy or therapeutic effect of innovative neurotherapies?

How concerned are you about the potential side effects of innovative neurotherapies?
