# Supplementary material for: Nurses' Attitudes Toward Innovative Neurotherapies in Memory Disorders: A Pilot Study
Source: CNS Neurosci Ther. 2026 Jul 17;32(7):e71015. doi: 10.1002/cns.71015 (PMC13377797; doi:10.1002/cns.71015)
Supplement: Supplementary file 3 — Table S1: Multiple linear regression analyses. [file CNS-32-e71015-s001.docx]

**Supplementary Table S1. Multiple linear regression analyses**

Regression analyses were performed in R version 4.4.1 (R Core Team. R: The R Project for Statistical Computing 2018. <https://www.r-project.org/> (accessed October 23, 2025).

In regression analysis reference categories were practical nurse (education), not at all familiar with innovative neurotherapies (familiarity), nursing home (workplace), <1 year (work experience) and male (sex).

Regression analyses for composite variables are summarized below. In tables β is unstandardized regression coefficient and SE is standard error.

# Perceived benefits

(benefit_score = β₀ + education + familiarity)

| Variable | β | SE | *p* |
| --- | --- | --- | --- |
| Intercept | 4.60 | 0.09 | <0.001 |
| Other education | -0.12 | 0.22 | 0.582 |
| Registered nurse | -0.08 | 0.13 | 0.549 |
| Student | -0.84 | 0.26 | 0.001 |
| Higher degree | 0.00 | 0.20 | 0.981 |
| Not very familiar with innovative neurotherapies | -0.03 | 0.12 | 0.831 |
| Somewhat familiar with innovative neurotherapies | -0.35 | 0.13 | 0.008 |
| Highly familiar with innovative neurotherapies | 0.44 | 0.40 | 0.283 |

Model fit: R² = 0.16, adj. R² = 0.11, F(7,113) = 3.05, p = 0.006

# Perceived barriers

(barrier_score = β₀ + education + familiarity)

| Variable | β | SE | *p* |
| --- | --- | --- | --- |
| Intercept | 4.47 | 0.09 | <0.001 |
| Other education | -0.32 | 0.21 | 0.141 |
| Registered nurse | -0.06 | 0.12 | 0.602 |
| Student | -1.04 | 0.23 | <0.001 |
| Higher degree | -0.09 | 0.18 | 0.610 |
| Not very familiar with innovative neurotherapies | -0.16 | 0.11 | 0.132 |
| Somewhat familiar with innovative neurotherapies | -0.27 | 0.12 | 0.030 |
| Highly familiar with innovative neurotherapies | 0.02 | 0.37 | 0.947 |

Model fit: R² = 0.19, adj. R² = 0.14, F(7,114) = 3.88, p < 0.001

# Enabling factors

(enabler_score = β₀ + education + familiarity + workplace)

| Variable | β | SE | *p* |
| --- | --- | --- | --- |
| Intercept | 4.63 | 0.08 | <0.001 |
| Other education | -0.02 | 0.18 | 0.896 |
| Registered nurse | 0.00 | 0.11 | 0.971 |
| Student | -0.72 | 0.22 | 0.002 |
| Higher degree | -0.15 | 0.17 | 0.369 |
| Not very familiar with innovative neurotherapies | -0.07 | 0.10 | 0.485 |
| Somewhat familiar with innovative neurotherapies | -0.22 | 0.11 | 0.047 |
| Highly familiar with innovative neurotherapies | 0.57 | 0.35 | 0.101 |
| Other workplace | 0.44 | 0.24 | 0.069 |
| Service housing/group home | -0.42 | 0.17 | 0.013 |

Model fit: R² = 0.24, adj. R² = 0.18, F(9,114) = 4.00, p < 0.001

# Professional role-related responsibilities

(score = β₀ + work experience + education)

| Variable | β | SE | *p* |
| --- | --- | --- | --- |
| Intercept | 5.05 | 0.28 | <0.001 |
| Women | 0.32 | 0.17 | 0.066 |
| 1–5 years | -0.90 | 0.26 | <0.001 |
| 5–10 years | -0.85 | 0.25 | 0.001 |
| 10–20 years | -0.81 | 0.26 | 0.003 |
| >20 years | -0.66 | 0.25 | 0.010 |
| Other education | -0.30 | 0.20 | 0.141 |
| Registered nurse | -0.18 | 0.12 | 0.151 |
| Student | -1.17 | 0.24 | <0.001 |
| Higher degree | 0.04 | 0.17 | 0.803 |

Model fit: R² = 0.22, adj. R² = 0.16, F(9,119) = 3.69, p < 0.001

# Worries

(worry_score = β₀ + work experience + education)

| Variable | β | SE | *p* |
| --- | --- | --- | --- |
| Intercept | 4.73 | 0.59 | <0.001 |
| 1–5 years | -1.71 | 0.62 | 0.007 |
| 5–10 years | -1.12 | 0.59 | 0.062 |
| 10–20 years | -1.80 | 0.62 | 0.005 |
| >20 years | -1.37 | 0.59 | 0.002 |
| Other education | -0.59 | 0.45 | 0.191 |
| Registered nurse | -0.46 | 0.29 | 0.123 |
| Student | -1.44 | 0.52 | 0.007 |
| Higher degree | -0.71 | 0.40 | 0.080 |

Model fit: R² = 0.16, adj. R² = 0.08, F(8,85) = 2.00, p = 0.057
